# Acute small bowel obstruction secondary to intestinal endometriosis, an elusive condition: a case report

**DOI:** 10.1186/1749-7922-5-27

**Published:** 2010-09-16

**Authors:** Alistair AP Slesser, Sufian Sultan, Faris Kubba, David P Sellu

**Affiliations:** 1 Department of Surgery, Ealing Hospital, Uxbridge Road, Southall, Middlesex, UK; 2 Department of Pathology, Ealing Hospital, Uxbridge Road, Southall, Middlesex, UK

## Abstract

**Background:**

Endometriosis is a benign condition affecting females of reproductive age. Although intestinal endometriosis is common it is rare for the condition to manifest as an acute bowel obstruction secondary to ileocaecal and appendicular endometriosis. This case is important to report as it highlights the diagnostic difficulty this particular condition presents to an emergency surgeon.

**Case presentation:**

We present the case of a 33 year old female of Asian origin who presented with symptoms and signs of an acute small bowel obstruction. A right hemicolectomy for suspected malignancy was performed with an ileocolic anastomosis. Histological examination demonstrated extensive endometriosis of the appendix and ileocaecal junction.

**Conclusion:**

Enteric endometriosis should be considered as a differential diagnosis when assessing females of reproductive age with acute small bowel obstruction. A high index of suspicion is required to arrive at a diagnosis of this elusive condition.

## Introduction

Endometriosis is a benign condition, affecting 4 to 17% of menstruating women. It has a peak incidence in the third and fourth decade. Its aetiology is unknown, although there is a high incidence in sterile females as well as in those who have a family history [1,2]. It is characterized by the presence of extra-uterine endometrial tissue. Endometriosis affects the intestine in 3 to 12% of cases and is generally an asymptomatic condition [1]. In rare circumstances, it can lead to obstruction requiring surgery. Clinically, the symptoms of bowel endometriosis are numerous and include abdominal pain, rectal pain, tenesmus, per rectal bleeding and constipation. Classically, the symptoms are worse during menses, but this is not always the case. This myriad of symptoms can make the condition difficult to diagnose acutely. We present a rare case of an acute small bowel obstruction secondary to ileocaecal and appendiceal endometriosis. This report serves as a reminder of this rare condition as well as highlighting the diagnostic difficulties it can pose.

## Case presentation

**Figure 1:**
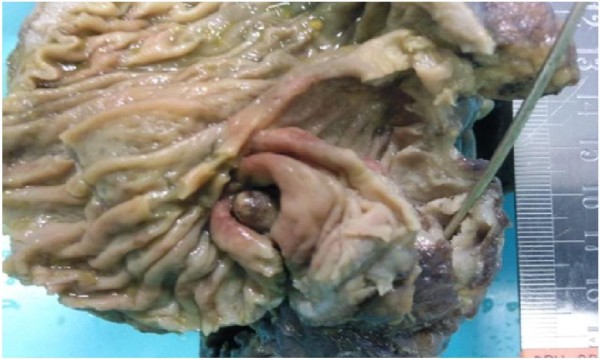
**macroscopic appearance of the resected specimen showing the caecal nodule**.

**Figure 2:**
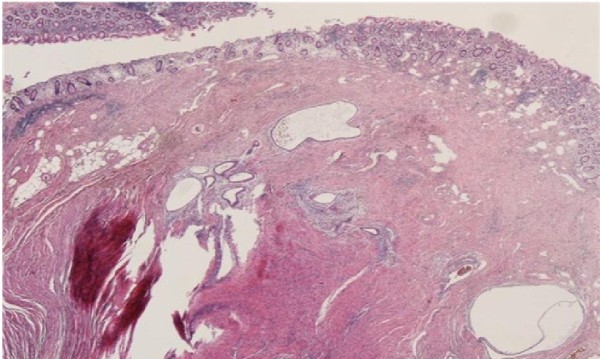
**microscopic appearance of endometriotic nodule in the submucosa comprising endometrial glands and surrounding stroma (magnification 20×)**.

A 33 year old woman of Asian origin was admitted to our Colorectal Unit with a one day history of absolute constipation and haematochesia. This was associated with a one week history of emesis that had gradually increased in severity. The patient was complaining of a one month history of generalised colicky abdominal pain. On the day of admission, the pain was described as severe and was scored as 10 out of 10. The constipation had commenced a month prior following her menses and had insidiously increased in severity. The patient's past medical history included three uncomplicated Caesarean sections and was otherwise unremarkable. At the time of admission, she was taking the oral contraceptive pill. She was a non-smoker and drank alcohol very occasionally. There was no family history of bowel cancer or inflammatory bowel disease. On examination the patient was comfortable at rest, haemodynamically stable and afebrile. Inspection revealed a distended abdomen with an obvious Pfannenstiel scar. On palpation, there was generalised tenderness with no rigidity or rebound tenderness. No herniae were found. Auscultation revealed tinkling bowel sounds. Per rectal examination demonstrated soft stool. Laboratory tests revealed a raised white cell count of 12700/mm3, a normal haemoglobin of 13.6 g/dL and an elevated C-reactive protein of 186 mg/dL. The arterial blood gas demonstrated a mild metabolic alkalosis with a pH of 7.461 and a base excess of 1.4. A urine dipstick and pregnancy test were both unremarkable. A supine abdominal radiograph showed dilated loops of small bowel. A CT abdomen/pelvis with oral and intravenous contrast was performed. This was reported as showing small bowel obstruction with a transition point at the terminal ileum which was thickened and stenosed. The CT appearances were suggestive of either Crohn's disease or Tuberculosis. The patient was treated conservatively with nasogastric suction and intravenous fluids. The patient initially responded well eventually regaining bowel function. However, the patient then suddenly redeveloped signs and symptoms of obstruction. Due to a rapid deterioration in the patient's condition a histological diagnosis could not be achieved prior to surgery. After obtaining informed consent from the patient, an emergency lower midline laparotomy was performed. Intra-operatively a dilated proximal small bowel was found with one constricting lesion affecting the ileocaecal junction which seemed to arise from the base of the appendix. The macroscopic appearances were suggestive of a malignancy. No other lesions were found. A right hemicolectomy was performed with a side to side stapled ileocolic anastomosis. Histological examination of the specimen was found to show a macroscopic the ileocaecal valve was compressed by outside mass and the mucosa showed an 8 mm fibrotic nodule occupying the appendiceal base which was on microscopy diagnostic of extensive endometriosis (see figures 1 &2). The patient made an uneventful post-operative recovery and was discharged. At outpatient follow up, the patient had not experienced any further symptoms and was well.

## Discussion

Interestingly, although intestinal involvement in endometriosis is common, it rarely causes acute intestinal obstruction [3]. The reported incidence of the involvement of different intestinal sites varies greatly in the literature, with the rectosigmoid colon, small bowel, appendix and caecum affected in 50-90%, 2-16%, 3-18% and 2-5% of cases [3]. It is postulated by Lin et al, that this is due to intestinal endometriosis being mainly an incidental finding [4]. It is clear, that as in our case, appendicular and ileocaecal involvement is rare. In a retrospective review of 7000 patients with endometriosis the incidence of caecal and appendix involvement was 4% and 3% respectively [5]. Similarly a twelve year study assessing the anatomical distribution of endometriosis found appendix and ileocaecal involvement in 6.4% and 4.1% of intestinal cases [6].

The aetiology of endometriosis remains unknown and controversial [2,7]. There are many theories but currently the most widely accepted theory is that of 'retrograde menstruation' causing the implantation and growth of endometriosis on the serosal surface of extra-uterine organs or occurring secondary to metaplasia in the pelvic peritoneum [2,5,8,9]. The concept of 'retrograde menstruation' is supported by the mainly pelvic distribution of endometriosis [6]. Although poorly understood, a combination of genetic aberrations as well as unknown environmental factors contribute to the development of endometriosis [9]. It is thought that the growth and invasion of endometrial tissue at ectopic sites is due to a process of neovacularization mediated by pro-angiogenic factors such as VEGF [10]. Small bowel endometriosis tends to only affect the bowel serosa and deposits tend not to be greater than 2 cm in size [1,3]. It is characterized by a patchy involvement of the bowel and macroscopically is 'grey glistening in appearance' [3]. Although generally asymptomatic, they can lead to local inflammation resulting in fibrosis and the formation of adhesions [1,11].In rare circumstances the disease can be more extensive, a histological review of fifty cases of intestinal endometriosis found that only 10% of intestinal cases had mucosal involvement [3,12]. Transmural disease damaging the mucosa can result in bleeding, the development of pseudo-tumours or obstruction secondary to 'stenosis' or 'kinking' [3,11]. The strictures and masses arise from a reactive smooth muscle hypertrophy secondary to disease present in the muscularis propria [3]. Rare cases of small and large bowel intussception, bowel perforation and malignant transformation have also been reported [11,13,14].

Acute bowel obstruction is a rare event occurring in less than one per cent of intestinal endometriosis and usually affects the rectosigmoid colon[1,15,16]. The case presented is rarely seen as small bowel obstruction only accounts for only 0.7% of all surgical interventions for endometriosis [16]. As our case serves to highlight, in an acute presentation the patient's history is unlikely to aid the diagnosis and thus it is unlikely for patients to be diagnosed pre-operatively [1-3,11]. It is a challenging condition to diagnose as small bowel endometriosis can manifest with acute and chronic symptoms that can mimic many different pathologies such as malignancy, inflammatory bowel disease, ischaemic colitis, infectious diseases and IBS [3,8,11]. Colicky abdominal pain is the most common presenting symptom of enteric endometriosis and is common to many other conditions such as Crohn's and is non-specific in cases of bowel obstruction [3,7]. Similarly, other common symptoms such as loose motions, constipation, nausea, emesis, pyrexia, anorexia and weight loss in isolation will not be diagnostic [3]. Haematochesia, such as was seen in our case is an uncommon symptom due to the low incidence of mucosal involvement [11]. The chronic symptoms of endometriosis tend to be 'pelvic pain, infertility, dysmenorrhoea and dyspareunia' [5,16]. Furthermore, the symptoms of bowel endometriosis can be associated with the patients' menstrual cycle in 18-40% of cases [2,7,11]. However, without a high index of suspicion these symptoms may not be elucidated or considered important particularly in an acute setting. This was clearly seen in our case, where the patient's symptoms had commenced following her menses and could have indeed aided our diagnosis. Laboratory tests such as CA125 are not sensitive enough for diagnostic use [8]. Contrast studies such as barium enemas may be helpful although they are falling out of favour and may not be specific [1,5]. As was evident in our case, cross-sectional imaging may not be helpful as it can be difficult to discern between ileal Crohn's and endometriosis [3]. Multislice CT with enteroclysis protocols can be useful in diagnosis as it may demonstrate focal or constricting bowel lesions [3,8]. MRI is currently the best imaging modality for enteric endometriosis with a sensitivity of between 77-93% [1,8]. If the condition is suspected then the urinary tract should be imaged, as an Urologist may be required [1]. Our case demonstrates that it is rare to be able to be solely reliant on imaging for the diagnosis of intestinal endometriosis [17].

Medical treatment with hormonal therapy such as OCP, Danazol or Gonatrophin antagonists can be attempted for intestinal disease when there is no obstruction [1,2,4]. This remains controversial as there are few reported cases of medical therapy being successful [1]. Indeed, in our case the patient's use of the OCP seemed to have no bearing on the progression of the disease. It is argued by some that the rare but potential risk of malignant transformation makes surgical resection manadatory [1]. When the surgery is elective then a laparoscopic approach should be favoured although it is important to explain the potential complications such as rectovaginal fistulae [18,19]. Surgery is only indicated in acute or sub-acute bowel obstruction that fails to resolve as well as in endometriotic tumours or when it is impossible to exclude a malignancy [11,14]. In an emergency setting, the main aim of surgery should be to relieve the obstruction. If the disease is suspected intra-operatively, then as many ectopic deposits as possible should be excised [1]. If there is a main bowel lesion then a resection margin of greater than 2 cm should be attempted [11]. However, our case helps demonstrate that it can be difficult to exclude a malignancy intra-operatively [3,20]. In such cases, it is appropriate to carry out an oncological resection. Post-operative hormonal therapy is advocated by some, however recent meta-analysis have failed to demonstrate any benefits [1,21].

## Conclusions

Acute bowel obstruction secondary to intestinal endometriosis remains a difficult condition to diagnose without an elevated index of suspicion. Endometriosis as a differential should be borne in mind when assessing females of a reproductive age who present with small bowel obstruction. A careful history may elicit symptoms related to the patient's menses and in conjunction with equivocal CT findings should raise the possibility of intestinal endometriosis. If the condition is suspected then a pre-operative MRI small bowel is indicated. Exclusion of bowel malignancy is essential and if in doubt an oncological resection should be performed.

## Abbreviations

CT: computed tomography; MRI: magnetic resonance imaging; OCP: oral contraceptive pill; VEGF: Vascular endothelial growth factor

## Consent

Written informed consent was obtained from the patient for publication of this case report and accompanying images. A copy of the written consent is available for review by the Editor-in-Chief of this journal.

## Competing interests

The authors declare that they have no competing interests.

## Authors' contributions

All authors contributed to researching, editing and writing the article. All authors read and approved the final manuscript.
